# Supplementary material for: Water Shortage Affects Vegetative and Reproductive Stages of Common Bean (Phaseolus vulgaris) Chilean Landraces, Differentially Impacting Grain Yield Components
Source: Plants (Basel). 2022 Mar 11;11(6):749. doi: 10.3390/plants11060749 (PMC8948600; doi:10.3390/plants11060749)
Supplement: Supplementary file 1 [file plants-11-00749-s001.zip › figure S1.pdf]

a

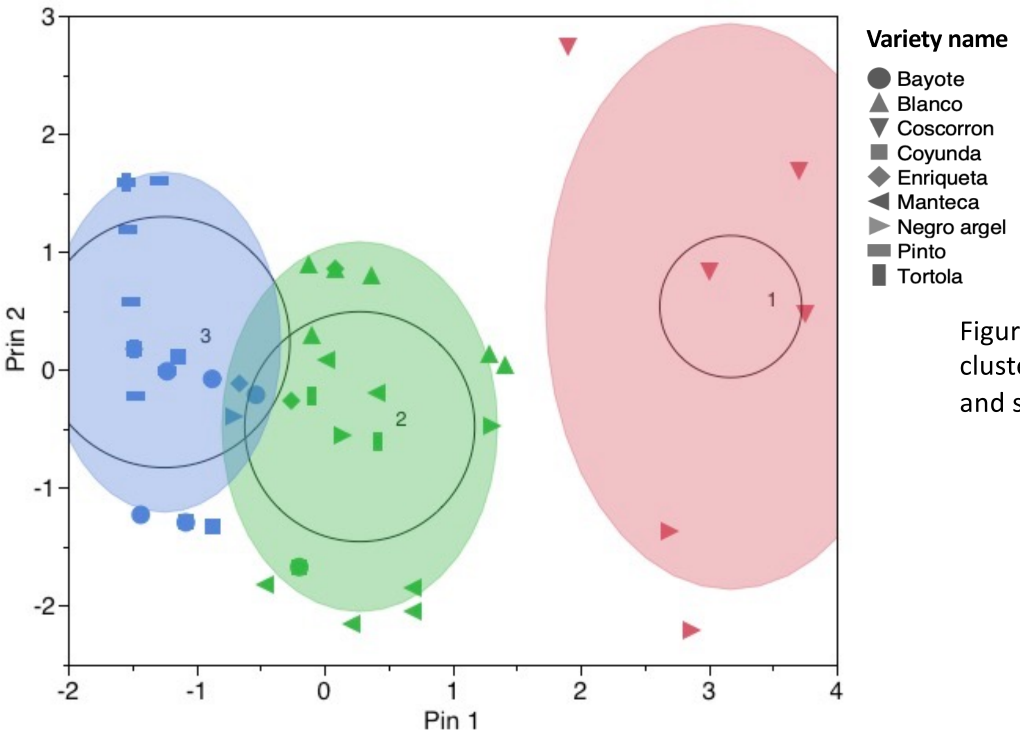

Figure S1. Phenological traits grouping for nine Chilean landraces. (a) Kmeans clustering analysis for traits flowering days, pod days, physiological maturity and seed filling (b) Scatterplot matrix for clustering from (a).

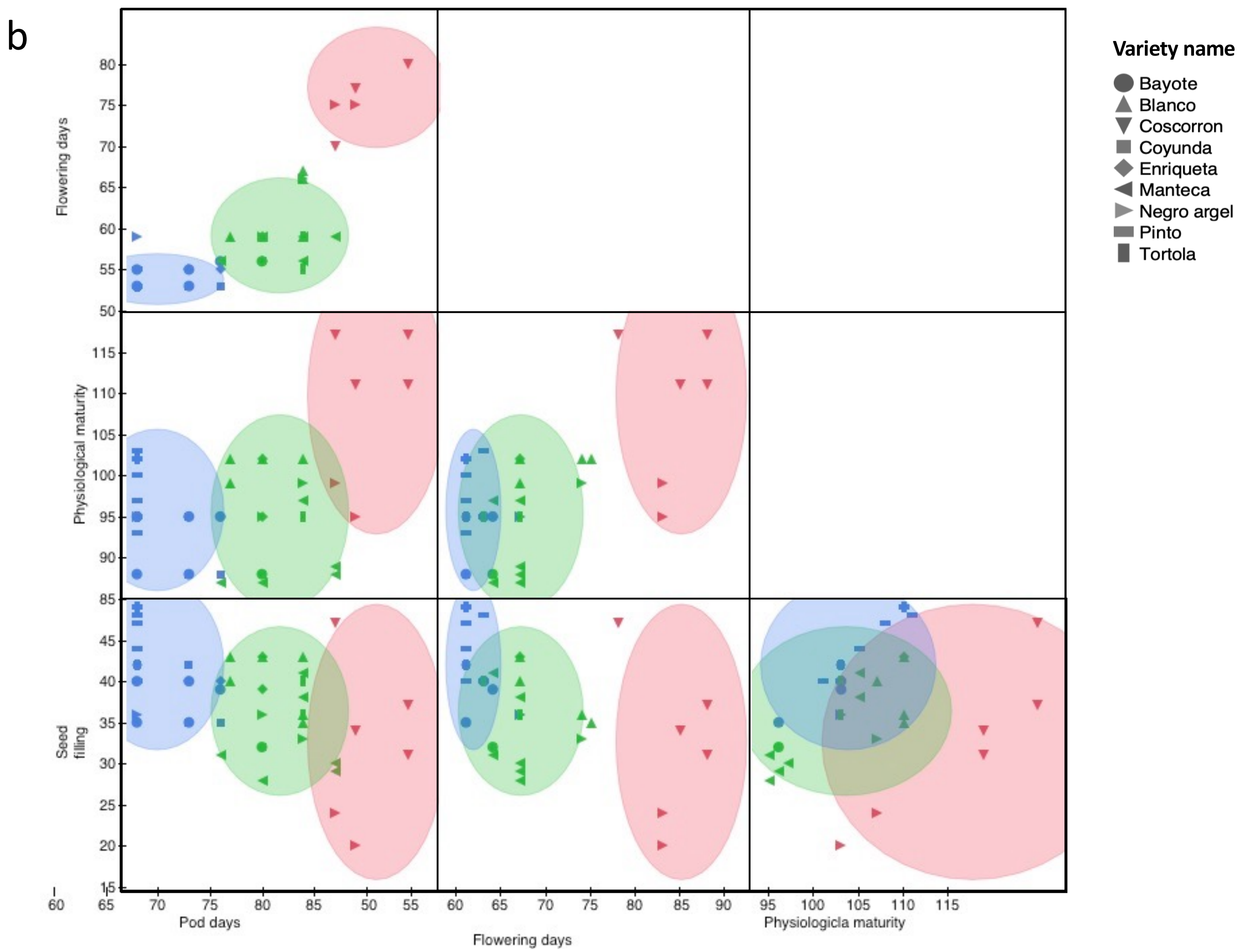
b
Flowering days
Physiological maturity
Seed filling
Pod days
Flowering days
Physiologicla maturity
Variety name
Bayote
Blanco
Coscorron
Coyunda
Enriqueta
Manteca
Negro argel
Pinto
Tortola
