# Supplementary material for: Water Shortage Affects Vegetative and Reproductive Stages of Common Bean (Phaseolus vulgaris) Chilean Landraces, Differentially Impacting Grain Yield Components
Source: Plants (Basel). 2022 Mar 11;11(6):749. doi: 10.3390/plants11060749 (PMC8948600; doi:10.3390/plants11060749)
Supplement: Supplementary file 1 [file plants-11-00749-s001.zip › Table S2.pdf]

Table S2. Water stress indices in Chilean landraces.

Landrace	DTI	GMP	MP
Enriqueta	1.9	470.3	517.4
Coyunda	2.1	491.4	545.5
Blanco	2.5	534.9	578.0
Pinto	1.4	400.3	420.3
Negro argel	1.1	358.7	396.5
Coscorron	2.5	535.7	554.4
Bayote	3.1	603.3	647.7
Tortola	1.6	433.8	487.9
Manteca	3.4	626.1	677.4
$p < 0.05$	***	***	**
LSD	0.9	106.8	128.0
Mean	2.2	494.9	536.1

** = $p<0.01$; *** = $p<0.001$
